# Unveiling the Role of Wetland Strategies in Antibiotic Risk Reduction across China by Machine Learning

**DOI:** 10.1021/acs.est.5c02866

**Published:** 2025-07-23

**Authors:** Lei Chen, Junxiang Shi, Danni Wu, Ying Zhu, Jonathan M. Adams, Jichun Wu, Xiaohui Chen, Hongyan Guo

**Affiliations:** † State Key Laboratory of Pollution Control and Resource Reuse, School of the Environment, 12581 Nanjing University, Nanjing 210023, China; ‡ Geomodelling and AI Centre, School of Civil Engineering, 120986 University of Leeds, Leeds LS2 9JT, U.K.; § State Environmental Protection Key Laboratory of Environmental Health Impact Assessment of Emerging Contaminants, School of Environmental Science and Engineering, 12474 Shanghai Jiao Tong University, Shanghai 200240, China; ∥ Key Laboratory of Surficial Geochemistry of Ministry of Education, School of Earth Sciences and Engineering, 12581 Nanjing University, Nanjing 210023, China; ⊥ School of Geography and Ocean Science, 12581 Nanjing University, Nanjing 210023, China; # Quanzhou Institute for Environmental Protection Industry, 12581 Nanjing University, Quanzhou 362046, China

**Keywords:** Data driven, Emerging pollutant, Wetland strategy, Environmental emission, Model interpretation

## Abstract

Pervasive antibiotic pollution in water environments has emerged as a serious threat to global ecosystem functions and public health. While wetland expansion—including protection, restoration, and construction, is widely promoted for sustainable water quality improvement, its effectiveness in mitigating antibiotic pollution remains poorly understood. Here, we develop a machine learning model based on a compiled data set of 337 experimental observations to quantify antibiotic removal and map risk distribution in wetlands across 2,833 counties/districts in mainland China. Between 2010 and 2020, the wetland area across China expanded by 34.7%, yet antibiotic removal improved by only 0.1%, failing to meaningfully reduce the risk. We find that antibiotic removal in wetlands is primarily constrained by input magnitudes rather than the wetland area. To address this, we proposed a multistage wetland management strategy to enhance antibiotic removal by 27.6% in 2020 and high-risk area reduction by 90.6% under optimal policies by 2035. Furthermore, we further identified the importance of wetland management strategies through an interpretable model. Our findings provide novel wetland strategy insights for policymakers and highlight the fact that wetland expansion without targeted management is insufficient for controlling antibiotic pollution, although it is an important cornerstone characteristic for water quality improvement.

## Graphical abstract

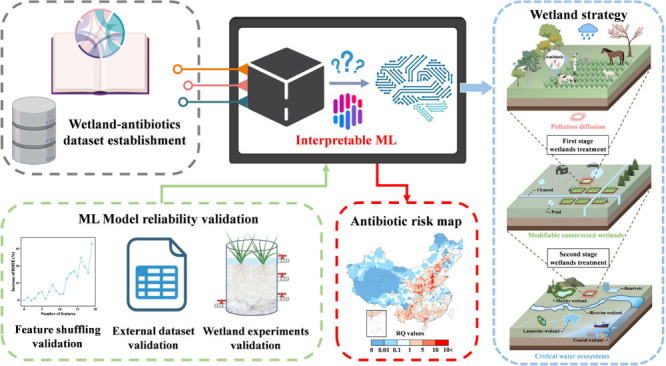

## 1 Introduction

The development and application of antibiotics greatly contribute to facilitating modern medicine, aquaculture, and agriculture over the past decades.^1^ Unfortunately, antibiotics are generally discharged or transported into the water environments causing antibiotic residue pollution, driven by widespread overuse, improper disposal, and inadequate wastewater treatment.^1,2^ Given the increase in human demand for animal protein, the antibiotic emission process to aquatic ecosystems is expected to persist in the future.^3,4^ Antibiotic residue can adversely affect the sustainability of ecosystems (such as antibiotic resistance and biotoxicity) even at low concentrations (ranging from ng L^–1^ to mg L^–1^), upgrading concerns about potential risks to global environmental and public health.^5,6^ Therefore, reducing antibiotic residue risks is important for maintaining the safety and sustainability of global water environments.

Wetland systems as strategic water ecosystem resources collect various environmental residual materials, providing a wide range of valuable functions and services for humans, such as water storage, resource recovery, biodiversity improvement, and water pollution control.^7–9^ The effectiveness of wetland systems has been demonstrated for antibiotic removal by a series purification mechanisms (for instance, sorption, biodegradation, and phytoremediation).^10–12^ Meanwhile, as part of the United Nations’ sustainable development goals, wetland expansion (including protection, restoration, and construction) has been currently recognized as a global priority.^13^ This expansion is a supplement in the ‘no-net-loss’ strategy of the wetland area.^14,15^ Between 2010 and 2020, the Ministry of Finance of the People’s Republic of China alone has spent over CN¥15.3 billion on wetland expansion, particularly through the National Wetland Conservation Plan and the Ecological Redline Program.^16^ Previous studies have identified that targeted wetland expansion can effectively remove nutrients and, in particular, reduce nitrogen contamination in aquatic environments.^14,17^ However, the effectiveness of this strategy on regional antibiotic pollution reduction remains poorly understood.

The current gap in understanding antibiotic removal through wetland strategies mainly lies with uncertainty over the contribution of the wetland to antibiotic removal at the regional scale. This further constrains the formulation of targeted wetland strategies aimed at reducing the antibiotic risk. Key questions persist: (i) can the current wetland expansion strategy effectively enhance regional antibiotic removal? (ii) How can we optimize future wetland strategies (e.g., wetland management) to achieve a substantial reduction in regional antibiotic risk? In this context, China stands out as a research priority, given its dual roles as one of the world’s largest antibiotic consumers and a party to the Ramsar convention.^4,17–20^

In this study, we focused on evaluating the effectiveness of wetland strategies in antibiotic risk reduction and providing optimized management approaches at multiple scales. We first quantified the impact of wetland expansion on antibiotic removal across China from 2010 to 2020 and mapped the spatial distributions of antibiotic risk in 2,833 counties/districts for 2020, with the assistance of machine learning (ML). Meanwhile, we assessed the risk reduction potential of targeted improvement practices (including wetland management, wetland expansion, and antibiotic use reduction) at the regional scale based on future scenario simulations. We further identified the role of a wetland area for antibiotic removal and derived actionable wetland management strategies at the individual wetland scale using interpretable ML. Our results provide informative insights into wetland strategies for antibiotic pollution reduction, which can help policymakers develop scalable wetland policies to achieve sustainable development goals and, as data availability grows, globally.

## 2 Materials and Methods

### 2.1 Data Collection and Preprocessing

The Web of Science database (http://apps.webofknowledge.com/) was utilized to obtain data on wetland for antibiotic removal by the comprehensive literature search with keywords, including “antibiotics” and “wetland”, up to June 2023. A total of 99 peer-reviewed studies were screened to establish the wetland-antibiotics data set with 337 data points retrieved and used (Supplementary Data-Excel 1). Nineteen features (16 numeric variables and 3 characteristic variables) were selected as the input labels of the ML model. The data-gathering process based on the following strategies and assumptions was incorporated to ensure the reliable data set creation. (i) All collected data were directly obtained from the text/table form listed in the literature or indirectly extracted from the figures.^21,22^ (ii) The antibiotic removal efficiency (ARE) as the prediction target of ML models was obtained from experimental data. For a research paper related to multiple antibiotic removal, the mean ARE was regarded as the output target, which was calculated by eq 1

1 $$Mean\;ARE=\frac{\overset{n}{\underset{i=1}{\sum }}c_{eff}^{i}}{\overset{n}{\underset{i=1}{\sum }}c_{\inf}^{i}}\times 100\%$$

where *c*_eff_^*i*^ is the effluent antibiotic concentration, *c*_inf_^*i*^ is the influent antibiotic concentration, and *n* is the number of antibiotic types.

For the missing data, we adopt two strategies to achieve missing data imputation: (i) we systematically reviewed other literature related to wetlands research from the same authors to find unrecorded values; (ii) the remaining missing values of variables were supplemented using the missForest method through R 4.3 software.^23^ The details of the missForest method are provided in Supplementary Text S1. The data set includes three characteristic variables: structural type, modified substrate, and aeration. Encoding methods (Text S2) including one-hot encoding were applied to convert characteristic variables to recognizable vectors. Moreover, the z-score standardization was used on the input features using StandardScaler in the Scikit-Learn package.^21,24,25^ The data distribution and variable explanations are shown in Supplementary Figure S1 and Table S2, respectively.

National wetland distribution and model input data at the regional scale were obtained from various sources and methods, including two China national land resource survey data sets (2010 and 2020) (https://gtdc.mnr.gov.cn/), the China Surface Climate Data set V3.0 (http://data.cma.cn/), the China National Environmental Monitoring Centre (https://www.cnemc.cn/), and a recently established veterinary antibiotic emissions data set in a best-guess emission scenario.^4^ These collection details can be found in Text S3. In addition, we systematically reviewed the peer-reviewed literature from ISI-Web of Science and the China Knowledge Resource Integrated Database (https://www.cnki.net/), using the search term ‘constructed purification wetland’ (specifically for wastewater treatment), in order to obtain a constructed purification wetlands list until 2020 (Supplementary Data-Excel 2) in China. Finally, a total of 1,161 constructed purification wetlands (CPWs) were collected from 558 papers, which is consistent with the results (nearly 1,200 until 2020) recorded by the Reports of China Wetland Research (https://www.cas.cn/jh/202212/W020221227354702998343.pdf).

### 2.2 ML Model Development

In the wetland-antibiotic data set, the total data points were randomly divided into the training section (80% points) and testing section (20% points) for ML model training and final evaluation, respectively.^26^ For the small data set, using relatively complex ML models (like neural network) may easily cause some potential issues, such as biased performance estimation, overfitting, and the lack of reliability and interpretability.^27,28^ Thus, we initially applied five basic ML algorithms, including extreme gradient boosting (XGBoost), K-nearest neighbors (KNN), random forest (RF), support vector regression (SVR), and KNN-Boost, to predict the ARE in this study. These ML models have been successfully applied in wetland spatial distribution and vulnerability assessment,^29,30^ management strategy simulation,^21^ and materials design optimization.^22^

During the training phase, each model was developed via comparing three hyperparameter optimization methods (grid search, random search, and Bayesian optimization). Meanwhile, the 5-fold cross-validation method was used to improve the capabilities of prediction and generalization for ML models by tuning the hyperparameters.^31^ In the 5-fold cross-validation process, the training data sets were further randomly split into pretraining and validation data sets in a ratio of 4:1. The coefficient of determination (R^2^) and root-mean-square error (RMSE) were chosen to assess and compare the prediction performance among the ML models.^21^

To explore the uncertainty of model final predictions, we applied a Monte Carlo-based bootstrapping method.^21,32,33^ First, we created an ensemble model by running 100 independent bootstrap iterations. We then calculated the mean value, standard deviation, and 95% confidence intervals for each model prediction ARE using a trained ensemble model. Meanwhile, we selected three validation approaches, including value shuffling, independent data set (Supplementary Data-Excel 1) validation, and wetlands experiment validation, to further ensure the model credibility. The details of the validation methods and wetland experiment setups are listed in Text S4.

Furthermore, the Shapley additive explanation (SHAP) approach based on cooperative game theory was utilized to identify the determining factors learned from ML and explain the underlying contribution of various features for wetland antibiotic removal. The SHAP method, widely used for key feature analysis, not only preserves the structure and predictive performance of ML models but also enhances model reliability through explanation.^34^

### 2.3 Antibiotic Surplus Calculations

The antibiotic surplus was calculated by the following equation

2 $$AS=AI-\sum {AR}_{i}$$

where AS is the wetland antibiotic surplus, AI is the wetland antibiotic input (from the antibiotic emissions data set), AR represents the wetland antibiotic removal, and *i* is the type of wetlands.

AR was estimated as a function of the ARE and AI, which determine the highest amount of antibiotics potentially available to the wetlands for removal. The ARE estimation of the wetland was calculated by the optimal ML model.

### 2.4 Scenario Simulations

According to the official plan, two major policies (wetland expansion and antibiotic use reduction) were considered for scenario simulations. Specifically, the restoration aims of future wetlands were to keep the overall area stable according to the National Wetland Conservation Plan (2022–2030) and the Protection and Restoration of National Key Ecosystems (2021–2035) released by the Ministry of Natural Resources of China. We selected three wetland restoration scenarios: WR1 (no expansion), WR2 (proportional placement with net expansion rate: 180 km^2^ yr^–1^), and WR3 (targeted expansion in high risk area with net expansion rate: 180 km^2^ yr^–1^). The calculation of the net expansion rate is based on the conclusion from the Reports of China Wetland Research: the net increase in the national wetlands area is 903 km^2^ between 2015 and 2020. The antibiotic reduction scenario is based on the requirements of the Action Plan for Veterinary Antibiotics Reduction (2021–2025), which specified more than 50% of farms to implement antibiotic reduction action by 2025. Thus, we assume that the proportion of farms participating in the antibiotic reduction action will increase by 10% yr^–1^ from 2020 to 2025 and then by 5% yr^–1^ until 2035 (100% farms participation by 2035). Meanwhile, simulations were conducted for three antibiotic abatement rates (including 20%, 40%, and 60%, Figure S2) in antibiotic reduction action. Finally, our future scenario simulation part includes 3 categories: wetland management scenarios (improved management-IM and no management-NM), wetland restoration scenarios (WR1, WR2, and WR3), and antibiotic use reduction (AR) scenarios (20%, 40%, and 60%), totaling 18 combinations.

### 2.5 Risk Evaluation

The risk quotient (RQ) method was commonly selected to estimate the ecotoxicity risk of a single antibiotic to aquatic organisms in receiving water bodies, based on the European Commission’s T*echnical Guidance on Risk Assessment* (2003).^35^ Due to algae being the most species impressionable to antibiotic toxicity stress, they were selected as sensitive species for assessing environmental risk.^35^ For evaluating the aggregate risk potential posed by multiple antibiotics, we employed the modified RQ method (eq 3) based on the “Cask Effect”^36^

$${RQ}_{total}=\frac{c_{surp}}{n\times {PNEC}_{algae‐\min}}$$ 3

where PNEC_algae‑min_ is the minimum predicted no-effect concentration (mg/L) of antibiotics discharged into wetlands using algae as the sensitive species, *n* represents the number of discharged antibiotic types, and *c*_surp_ indicates the antibiotics concentration surplus (mg/L) calculated by eq 4 according to the prediction results of the developed ML model.

$$c_{surp}^{i}=c_{i}\times \left(1-{ARE}_{i}\right)$$ 4

Here ARE_*i*_ represents the prediction of the ARE in the *i* type of the wetland at the county-scale, and *c*_*i*_ was estimated using eq S1 in Text S3.

In this study, a total of 80 antibiotics were included in the antibiotic emission inventory, of which Tiamulin was recorded as the antibiotic with the minimum PNEC_algae_ value (3 ng/L).^4^ Here, the high-risk county area (HRA), namely, the county area including high-risk natural wetlands, was selected as a regional risk assessment indicator. This RQ estimation method may be more rigorous than the previous evaluation method, which is the total RQ value obtained by cumulative RQ of individual antibiotics.^37,38^

## 3 Results

### 3.1 Model Performance and Validation

Through comparison with the R^2^ and RMSE results for each ML model, the XGBoost model presents the best prediction performance (Figure 1a and Figure S3), in which the train and test R^2^ values were 0.92 and 0.84, individually, while the RMSE values were 6.67 (training) and 9.28 (testing). There is no situation of acute overfitting for the XGBoost model since the gap value is below 0.15 between train and test R^2^.^25^ Additionally, the tree-based ML model, as a subcategory of supervised ML methods, demonstrated the ability to resist overfitting and counter-noise features, especially when working with small data sets.^25,39^ Therefore, the XGBoost model was selected as the best-performing algorithm for predicting the ARE of wetlands.

**Figure 1:**
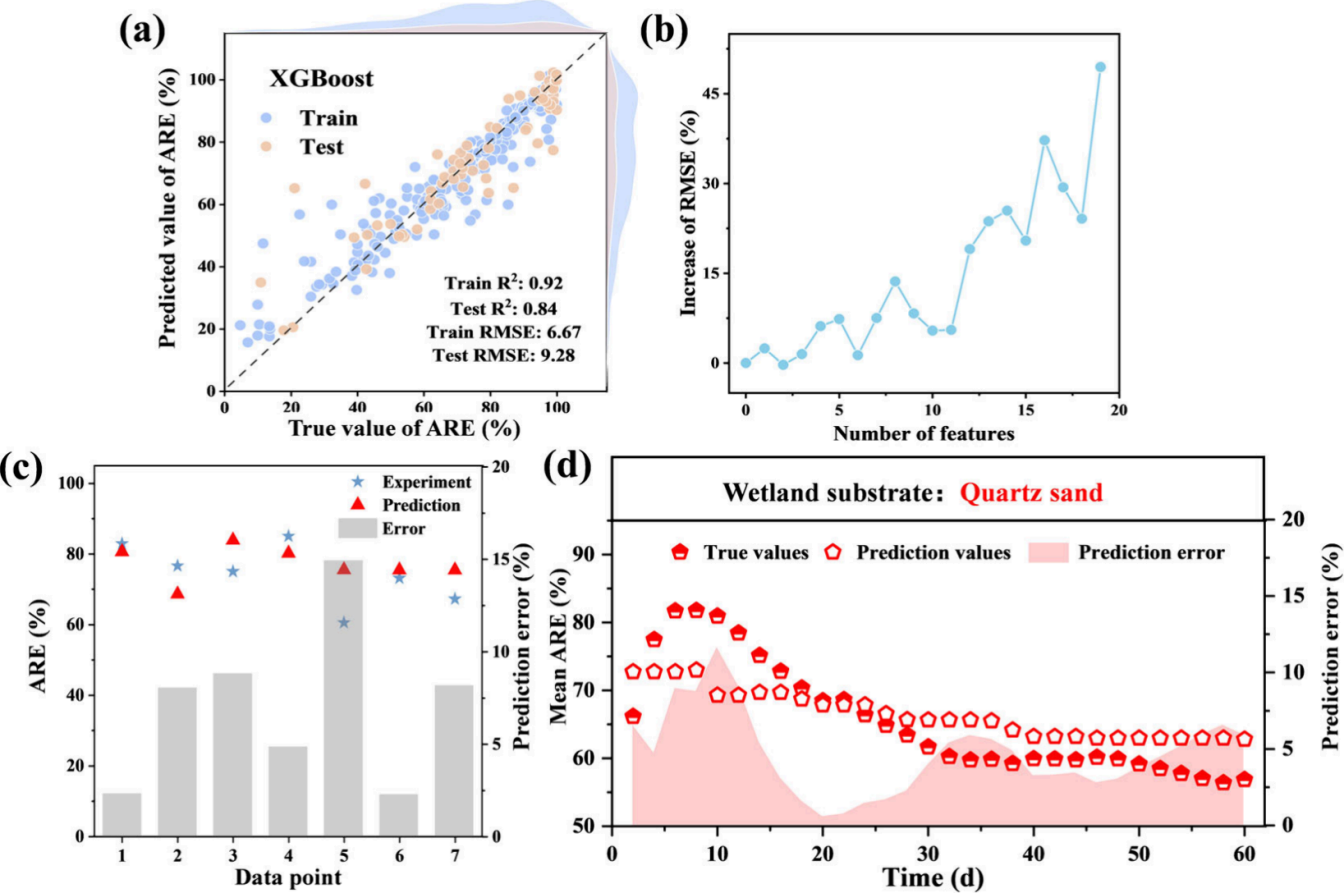
(a) Prediction performance of XGBoost and (b–d) model reliability verification via feature value shuffling, independent data set, and CPWs experiments, respectively.

The robustness of model prediction should be validated internally and externally.^40^ As shown in Figure 1b, the model prediction performance was significantly disturbed after feature shuffling, which indicated the XGBoost prediction model developed in this study has certain reliability.^40^ The results of external data set validation (Figure 1c) demonstrated that the prediction errors of the model were lower than 15%, indicating the accuracy of our developed model.^22^ In established wetland experiment validation (Figure 1d), the model prediction error of all test points is less than 12% (mean prediction error = 4.7%). It should be emphasized that this developed model in this study is closer to forecast the gross estimation of antibiotic removal performance in wetland rather than the precise dynamic simulation. Thus, the level of prediction error was acceptable.

### 3.2 Wetland Expansion Fails to Significantly Remove Antibiotics

Between 2010 and 2020, China made notable enhancements in wetland restoration (Figure 2a and Figure S4a). The areas of natural and constructed wetlands increased by 47.85% and 5.93%, respectively. In general, wetlands are unevenly distributed across China. In 2010, the highest wetland densities in China were found along the eastern coast (from the Shandong province to Shanghai), the central Yangtze River basin (Hubei province), and the southern coast (Guangdong province), and these were still consistent wetland hotspots in 2020 (Figure 2a and Figure S4a). We used the optimized XGBoost model for quantifying the changes in antibiotic removal capacity of wetlands caused by wetland restoration from 2010 and 2020 (Figure 2b and Figure S4b), assuming no environmental variation. Our results suggest that wetland antibiotic removal masses were estimated as 19.37 kt yr^–1^ (95% confidence interval, 17.37–20.25 kt yr^–1^), a total that represents approximately 72.23% of the antibiotic emissions across China in 2020, which are only 202.86 t yr^–1^ higher than the 19.17 kt yr^–1^ (17.00–20.17 kt yr^–1^) removal in 2010 (Figure 2b and Figure S4b). Traditionally, regions with a high wetland area have the potential for high contaminant removal, since wetland environments are rich in plants and microorganisms, making them hotspots for pollutant removal.^8,12^ However, the wetland area tells only part of the story. The magnitude of actual antibiotic removal is inherently dependent on the amount of antibiotic input in the wetland catchment area.^14^ As shown in Figure 2e, the top five areas for wetland restoration percentage-Qinghai (43.2%), Gansu (32.6%), Inner Mongolia (32.4%), Tibet (31.2%), and Heilongjiang (28.6%)—were responsible for 88.3% of national wetland restoration but only accounted for 7.8% of national antibiotic emissions, which indicates a clear disconnect between wetland restoration sites and antibiotic emission source zones over the decade. Additionally, relatively high removal enhancement regions included the following: the Henan, Shandong, Gansu, Guangdong, Guangxi, Yunnan, Hunan, and Hubei provinces of China (Figure 2e). The total wetland area of these provinces, however, did not significantly change (below 2.2%) between 2010 and 2020 (Figure 2e). Accordingly, enhanced removal of antibiotics in these provinces may be attributed to internal wetland management optimization (including detailed changes in the wetland distribution and types). In brief, wetland ecosystems showed high but not maximum capacity for antibiotic removal in China due to the limitations from antibiotic input variance and wetland management patterns at present.

**Figure 2:**
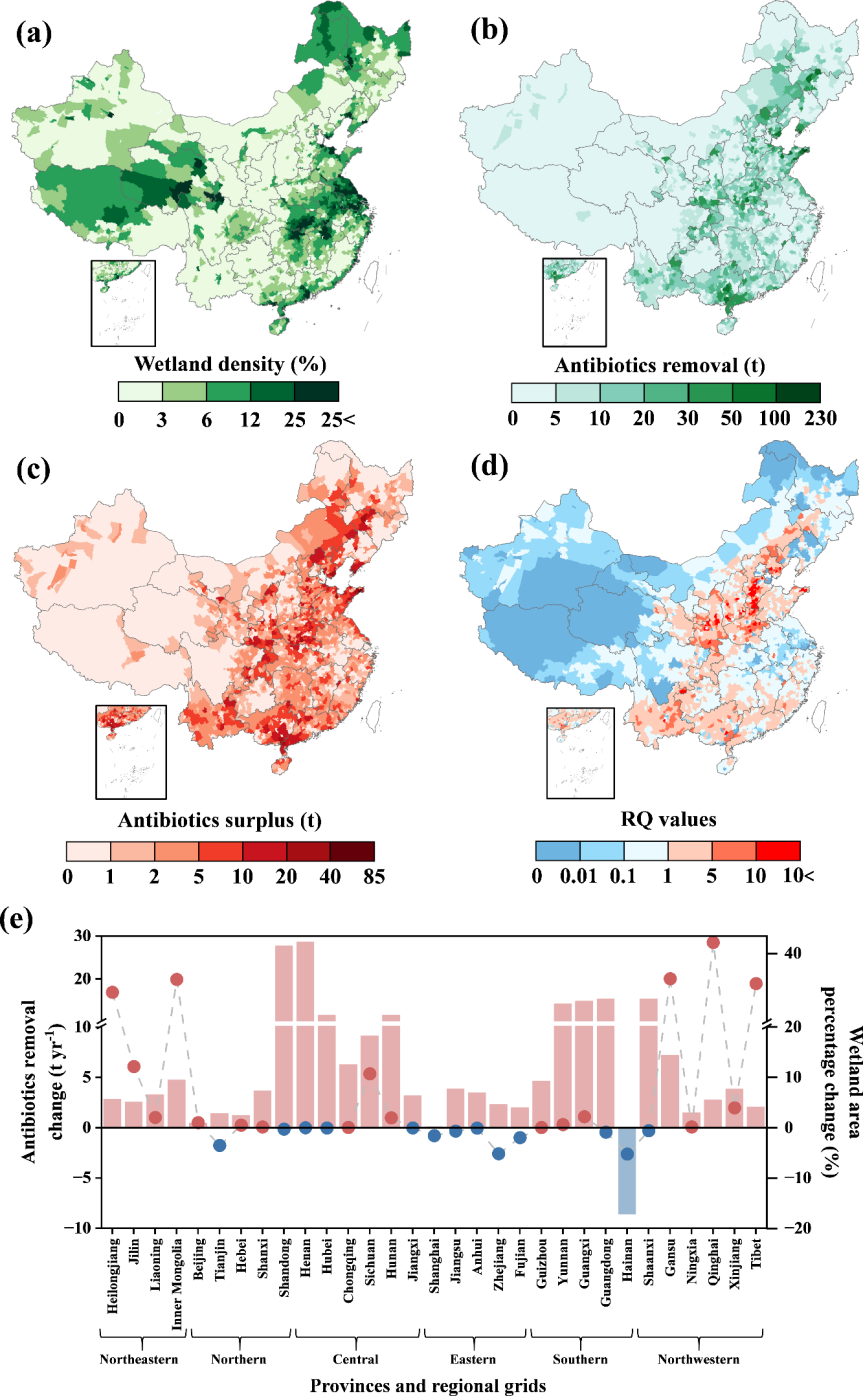
Spatial distribution of (a) wetland density, (b) antibiotic removal, (c) antibiotic surplus, and (d) RQ across China (the county level) in 2020, respectively. (e) Provincial changes in antibiotic removal (bar) and wetland area percentage (line) from 2010 to 2020. Note that for the color changes, the red and blue colors mean positive and negative changes, respectively.

The region with high levels of antibiotic surplus generally has relatively high ecological risk (Figure 2c,d and Figure S4c,d). Generally, the spatial distributions of antibiotic removal, surplus, and risk remained relatively consistent with regional antibiotic emission magnitudes.^4^ The high-risk sites (RQ value > 1) are currently concentrated in the Hebei, Henan, Shandong, Shanxi, Shaanxi, Chongqing, Yunnan, Guangxi, and Guangdong regions of China (Figure 1c). According to our estimates, the antibiotic surplus concentrations in wetlands fell within the range of 0.0–19.3 μg L^–1^ (n = 2837, Figure 2c) in 2020 across China, with median and mean concentrations of 162.6 and 443.1 ng L^–1^, respectively, which are comparable to the antibiotic concentration (ng L^–1^-μg L^–1^) detected in surface water from other recent studies.^41,42^ According to the map of RQ values (Figure 2d), we can identify regions (over 2.1 × 10^6^ km^2^) for improvement on the current state. Notably, wetland restoration, mainly concentrated in the northeastern and northwestern regions (Figure 2e), slightly reduced the antibiotic risk level (from medium/low risk to low/no risk; see Figures 2d and S4d) despite the antibiotic removal magnitude only increasing by 30.70 t yr^–1^ over the decade in these provinces (Figure 2e). According to the RQ calculation method, the RQ values were directly related to antibiotic concentration, while the concentration value is negatively correlated with a greater presence of a regional wetland area. The findings further underscore the importance of continuous restoration or protection of wetlands as the cornerstones for controlling risk, regardless of antibiotic removal magnitudes.

### 3.3 Antibiotic Removal Enhancement by Improved Wetland Management

Most natural wetlands and reservoirs are managed to conserve biodiversity, restore degraded water ecosystems, and store water, rather than often serving as wastewater treatment due to the diversity of practical objectives.^12,43^ Meanwhile, our analysis described above suggests that the wetland catchment areas with high-antibiotic input have great potential to maximize water quality benefits. To meet practical user needs and test our hypothesis, we further used the ML model to simulate an improved wetland management measure scenario—multistage wetland treatment: through human intervention, the regional antibiotics were preferentially treated by constructed wetlands (excluding reservoirs) and then evenly allocated to the remaining wetlands (including natural wetlands and reservoirs) as the second-stage treatment units within the region for further purification (Figure 3a). This approach allows for the catchment of antibiotics in select constructed wetlands, reducing the concentration limitation related to antibiotic removal performance and further alleviating risk in critical wetland ecosystems.

**Figure 3:**
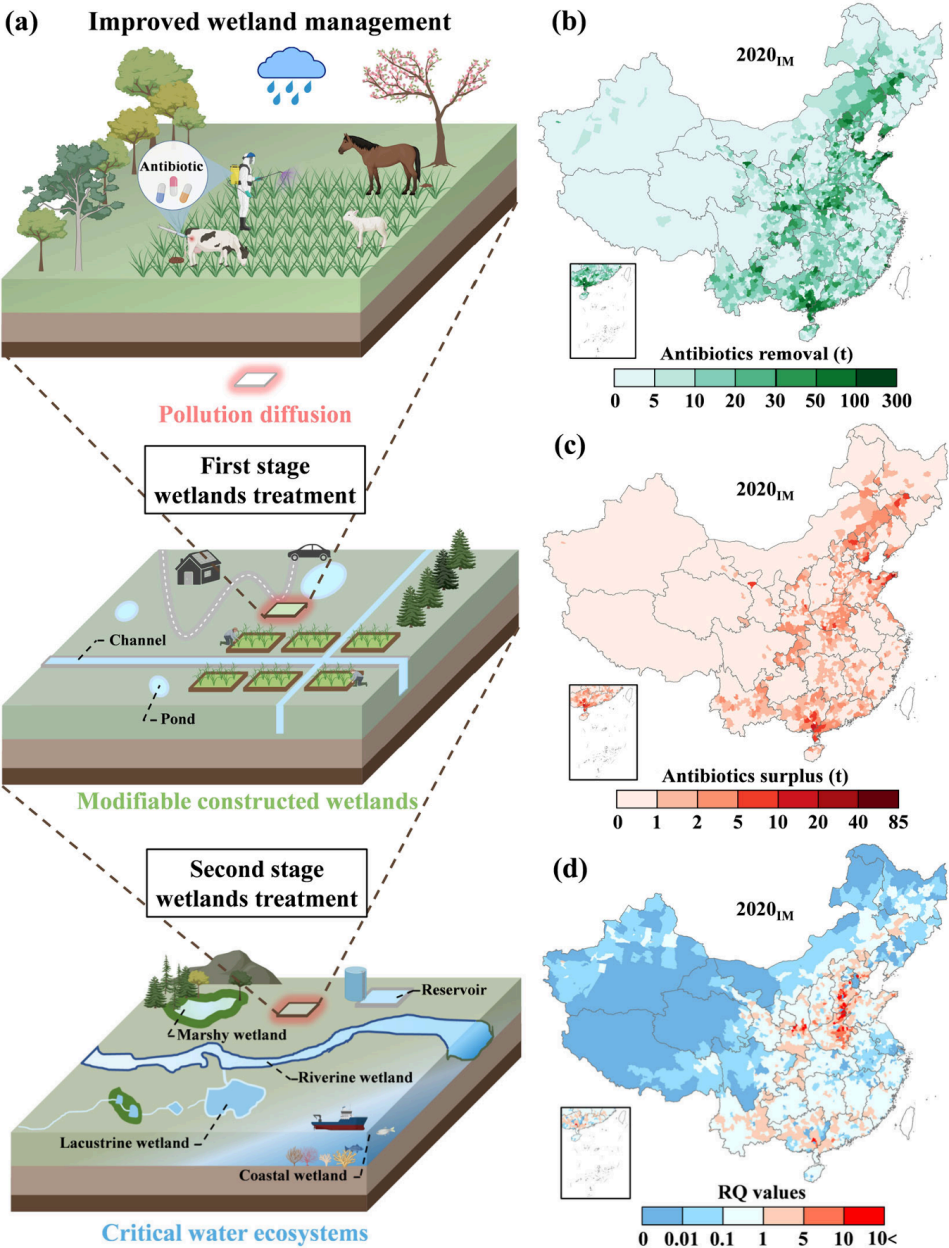
Influence of improved wetland management for antibiotic removal enhancement. (a) Schematic diagram of the IM. (b-d) Antibiotic removal, antibiotic surplus, and RQ distribution under the IM scenario across China in 2020, respectively.

Our results indicate that for this optimized wetland management scenario in 2020, total antibiotic removal is increased by 27.6% (Figure 3b), constituting an additional 5.35 kt (4.77–5.58 kt) antibiotic removal across China, and total antibiotic surplus decreased by 71.9% (Figure 3c), compared to the unmanaged scenario (Figure 2b, c). This quantitatively demonstrates the value of the modified wetland management approach. With a wetland management approach improved for antibiotic removal, we find that considerable strides can be taken toward achieving policy aims for antibiotic risk reduction in relatively critical water ecosystems (e.g., natural wetlands and reservoirs), providing an approximately 55.6% decrease in the total area of high-risk counties (Figure 3d). Of course, every regional situation varies. It should be emphasized that there are still some areas (about 9.33 × 10^5^ km^2^) where high-risk watersheds exist, and even part of them have increased antibiotic risk (for example, Shenzhou city and Guantao county of Hebei Province) despite adopting the enhanced wetland management strategy. This is expected because these regions all include two apparent features: high antibiotic emissions and low wetland areas. Accordingly, besides optimizing wetland management, appropriate policies such as targeted wetland restoration and antibiotic use control are necessary to strengthen antibiotic risk reduction,^8,14^ which are in line with the requirement of official policies after 2020 in China, namely, the National Wetland Conservation Plan (2022–2030) and the Action Plan for Veterinary Antibiotics Reduction (2021–2025).

### 3.4 Future Scenarios

To understand the potential future risk trajectories through China’s policies influence, we projected the fluctuation of the HRA caused by measure variations, including the IM, WR, and AR, from 2021 to 2035 using the XGBoost model. This approach allows us to anticipate risk degrees under diverse scenarios, enabling policymakers to gain insights into the potential contribution of different measures and make judicious decisions.

For all 18 scenarios, the HRA decreases almost linearly over 15 years (Figure 4a), with the highest reduction degrees being seen for the IM-WR3–60 scenario. Under the IM-WR3–60 scenario, the HRA is predicted to decline by 90.6% (about 1.91 × 10^6^ km^2^) by 2035. To gain a deeper understanding of the relative contribution related to the different measures decreasing the HRA, we utilized the control variable method to analyze the mean HRA reduction efficiency for all individual measures over 15 years (Figure 4b). Among the three types of risk reduction measure, the IM had the maximum potential for the HRA reduction (49.8%, 43.6–55.2%), followed by the AR (5.6–31.0%), and the WR (0.2–7.6%). For the AR, it is evident that a higher AR level is in favor of the HRA reduction since reduced antibiotic consumption can directly weaken antibiotic emission at the source.^44^ Meanwhile, spatial targeting of wetland restoration (WR3, 4.1%) was more effective in the HRA reduction than nontargeted expansion (WR2 measure, 2.5%), which aligns with previous studies.^8,14^ It is crucial to acknowledge that restoring wetlands and antibiotic use reduction have been integral in controlling antibiotic risk although their contribution is below that of the IM measure,^45,46^ as adequate wetland volume and low antibiotic input can guarantee the stability of risk reduction via IM (Figure 3d) and the sustainability of further decreasing the HRA in the future (Figure 4a).

**Figure 4:**
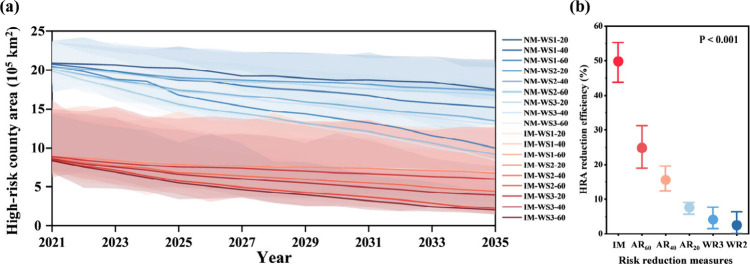
Future scenarios and measure contribution. (a) HRA (The shaded area represents the 95% uncertainty in scenario simulations) and (b) Mean HRA reduction efficiency of each measures between 2021 and 2035 (The P value was calculated with a one-way analysis of variance).

### 3.5 Wetland Management at the Field Scale

Specifically, it is also critical to strengthen risk control by improving the engineered wetlands at the individual wetland scale besides watershed-scale effects.^12,14,47^ Constructed purification wetlands (CPWs, alternatively known as treatment wetlands) have been applied in over 50 countries to improve water quality for decades.^12^ Thus, we first evaluated the application and distribution of field-scale CPWs in China. There are currently 1161 field-scale CPWs, with a total area exceeding 540 km^2^, in China for water purification (Figure 5a). The majority of these CPWs is located in the eastern (41.1%) and northern regions (26.3%), followed by the southern (13.5%) and central provinces (9.6%) (Figure 5a). Despite an impressive increase in field-scale CPWs (only 366 systems about 174.4 km^2^ by 2010, Figure S5) across China from 2010 to 2020, the coverage of these facilities remains at a low level at present, accounting for only 0.55% of constructed wetlands (excluding reservoirs). In the future, improved multistage wetland treatment can evolve into a three-stage process with widespread CPWs adoption.

**Figure 5:**
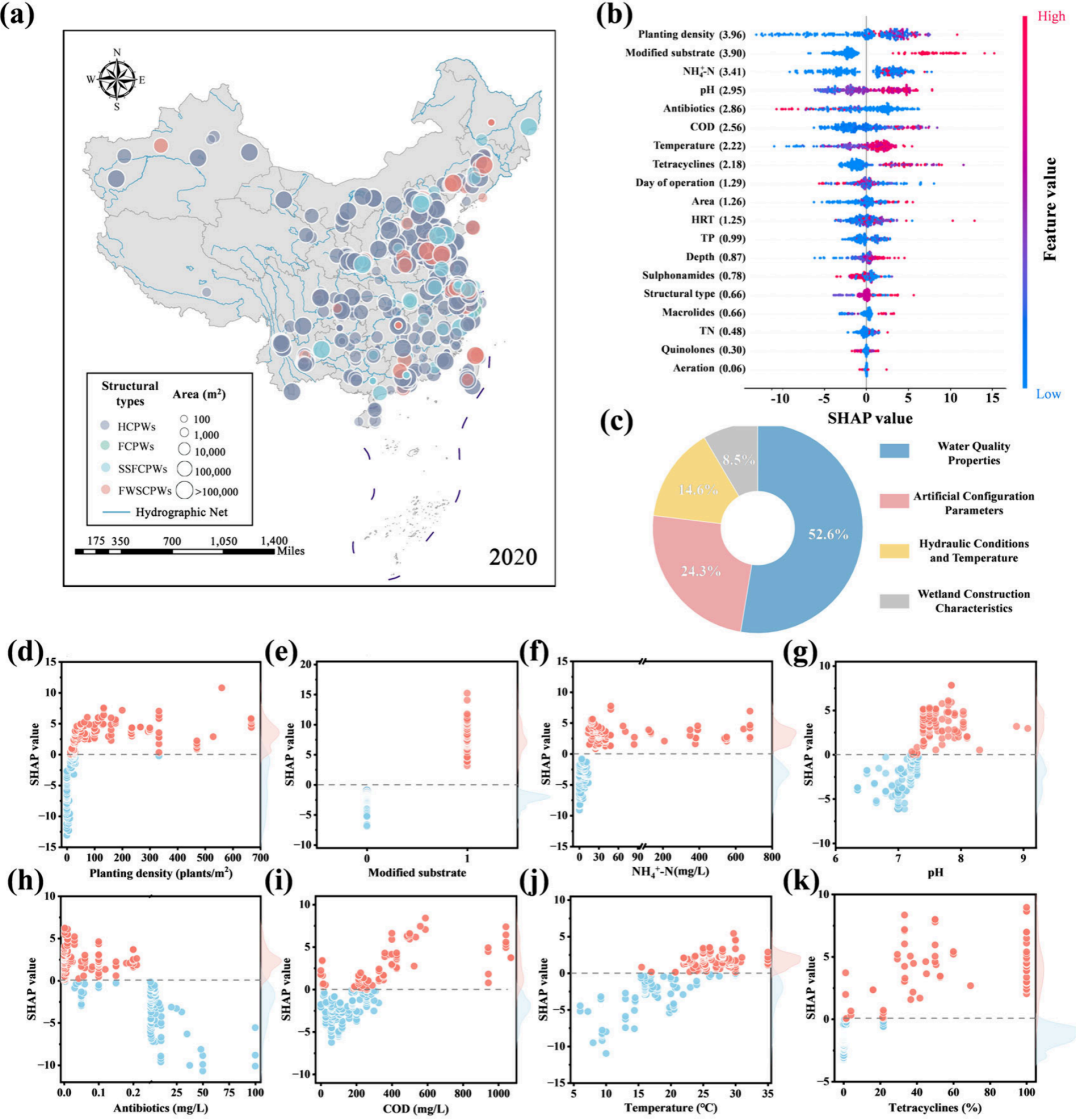
Distribution of field-scale CPWs across China and feature importance. (a) National distribution of field-scale CPWs for water pollution control; (b) SHAP summary plot for each input feature with absolute mean SHAP values; (c) The relative contribution of the four categories of input features for the prediction (output); The SHAP scatter plots of the top eight features: (d) planting density, (e) modified substrate, (f) NH_4_^+^-N concentration, (g) pH, (h) antibiotic concentration, (i) COD content, (j) temperature, and (k) percentage of tetracyclines. The gray dashed line is the demarcation line of the SHAP value (SHAP value = 0). The red point demonstrates that the influence of parameters is positive, while the blue point represents the negative effect for model prediction.

Despite decades of application, the successful and sustainable application of CPWs remains like a black box, with contaminant removal performance depending on a variety of factors, for example, water quality properties, artificial configuration parameters, and so on.^12,48^ Accordingly, we further explored potential influences linking these variables to the ARE of CPWs using interpretable ML, providing achievable intensification strategies for advancing the broader application of CPWs. The SHAP method can reveal the decision-making process of ML models and provide quantitative insights into the relative importance of variables on estimates of the ARE.^34,49,50^ Water quality properties had the highest overall SHAP values (accounting for 52.6%), affecting more of the variation in the ARE of CPWs than artificial configuration parameters, hydraulic conditions, and temperature and wetland construction characteristics (Figure 5c). High planting density and modified substrate were dominant influencing factors for higher estimates of the ARE (Figure 5b-e). As two of the main structures of CPWs, their important role seems to be reasonable, since the plant uptake, substrate adsorption, and biodegradation supported by them are the main routes for antibiotic removal in CPWs.^12,51^ However, when the planting density was extremely high (>100 plants/m^2^), the SHAP values were kept relatively constant, indicating that strengthening the ARE is finite through configuring a higher planting density (Figure 5d). The lack of the adsorption sites of microorganisms and plants caused by low-concentration nutrients can weaken the ARE of CPWs,^52^ aligning with the observed trend of SHAP values (Figure 5f, i). Moreover, SHAP contributions demonstrated that suitable pH (7.2–8) and temperature (25–30 °C) were associated with higher ARE estimates (Figure 5g, j), as an appropriate growth environment for microbial communities avails increasing bioactivity and antibiotic removal performance.^53^ Antibiotic concentration, along with the proportion of tetracyclines, showed a clear directional influence on estimates of the ARE, with lower antibiotic concentration and higher tetracycline percentage contributing to a higher ARE in CPWs (Figure 5h, k). Previous studies have identified that tetracyclines are susceptible to photodegradation compared with sulfonamides and macrolides.^52,54^

Notably, antibiotic removal equals the product of the ARE and antibiotic concentrations (Materials and Methods), so that the low ARE displayed at high concentrations does not mean low antibiotic removal magnitudes in wetlands (Figure 5h). Meanwhile, the importance of the wetland area (mean SHAP value = 1.26) only ranked tenth (Figure 5b), further suggesting the finiteness of wetland expansion for antibiotic removal. The permutation importance analysis by Eli5 (version 0.13.0) was further used to reconfirm the SHAP value results. This model agnostic method was utilized to judge the feature’s importance of the ML model via evaluating the influence of changes in each feature adding or discarding on model prediction accuracy.^22^ The results showed that the importance of variables remained relatively consistent (Figure S6), which demonstrated that the analysis based on SHAP is reliable.

Our study findings underscore the pivotal role of water quality properties and artificial configuration parameters on antibiotic removal in CPWs, which is line with previous research.^10,55^ However, it is relatively difficult to improve the ARE by optimizing water quality properties, due to the uncontrollable characteristics of influent quality and the constraints of economic factors. Specifically, even though wetland constructors understand that suitable pH favor increasing the ARE in wetlands, the properties of actual wastewater are hard for decision-makers to control, and in addition, it is also challenging to maintain a suitable pH due to economic considerations. Accordingly, three actionable and brief counsels are given as follows: (i) Decisionmaker can improve the ARE of CPWs by substrate modification; (ii) The suitable range of planting density is from 30 to 100 plants/m^2^, and (iii) The hybrid or subsurface flow CPWs with a high ARE are advised as the structural type (Figure S7). Based on this counsel, we further simulate the influence of enhanced constructed wetlands composed of channels and ponds (50 plants/m^2^ and modified substrate) on the HRA in the IM-WR3–60 scenario by 2035 (Figure S8). The result showed that the HRA is further decreased by 29.5% through enhanced wetland management at the individual wetland scale. Our findings highlight the significance of improved wetland management for reducing antibiotic risk, regardless of whether at the regional or individual wetland scale.

## 4 Discussion

As a party to the Ramsar convention, China is obligated to reduce potential risk effects of antibiotic emissions on wetland ecosystem services (like biodiversity and sustainability) via a series of policy interventions and practices, including continuous wetland restoration and decreased antibiotic consumption, which have been identified as the cornerstones for achieving risk control.^4,8,14,19^

Our results pointed out that the period from 2010 to 2020 witnessed a significant improvement in overall wetland coverage by Chinese restoration practice. However, the wetland restoration measure alone did not notably improve antibiotic removal and HRA reduction across China. This is different from the traditional understanding about improving significant removal of other pollutants (like nitrogen) through wetland area expansion.^14,17^ The main reason for this divergence is that the environmental mass input of antibiotics (as emerging contaminants, approximately 26.83 kt yr^–1^ across China in 2020) is much lower than that of nitrogen (million tons level).^14,56^ As a result, we provided an IM strategy (multistage wetland treatment) at the regional scale for addressing this limitation. In light of our analysis, this approach can achieve a significant antibiotic removal enhancement of over 27% by 2020, leading to a substantial reduction (about 1.17 × 10^6^ km^2^) in the HRA. Nevertheless, it is important to acknowledge possible adverse outcomes caused by the inherent regional variations, such as the increased risk for some counties. This spatial discrepancy could be attributed to the scarcity of ‘cornerstone’ characteristics (e.g., adequate wetland density and/or low antibiotic discharge) in partial regions. Therefore, we advocate for this IM strategy, coupled with targeted wetland restoration and a high reduction in antibiotic use. Collectively, the national HRA reduction under this comprehensive policy combination (IM-WR3–60) will materialize by 90.6% by 2035. Additionally, we further considered wetland management at the individual wetland scale for antibiotic removal, particularly the improvement of single wetland performance by three actionable practices. Although some recommendations have been confirmed by previous studies,^10,12,51,55^ we balanced individual differences between various studies and further confirmed these conclusions’ reliability using interpretable ML from a comprehensive perspective for the first time.

Notably, findings in this study may be affected by the distribution of data across the wetland system scale in the model development.^57^ Of the wetland-antibiotics data set used in our model development, only 12.7% were classified as field-scale wetlands (area > 2 m^2^). Further research is needed to assess the potential influence of data limitation and minimize modeling uncertainty. This involves expanding field-scale wetland data collection, standardizing methodologies, and improving data quality.^21^

## 5 Implication

Our study offers valuable insights for policymakers and researchers: traditional wetland expansion strategies need to be combined with wetland management strategies for enhancing antibiotic risk reduction within China’s wetland ecosystem. Meanwhile, we acknowledge that more extensive monitoring data and long-term field experiments are also important for improving the reliability of wetland strategies. This strategy may be applicable to the risk control of other emerging pollutants with low environmental emissions, because the mechanism of pollutant removal in wetlands is connected to some extent. In addition, it is important to emphasize that these risk solutions are not ‘one size fits all’, as they are also intertwined with economic conditions, historical context, and popular attitudes. Future strategy implementation should be targeted and tailored to actual regional disparities. Nonetheless, we remain steadfast in our belief that the current results create an important context for dialogues and discussions in the wetland protection field and in response to the challenges posed by emerging pollutants.

## Supplementary Material







## Notes

Data imputation method – missForest (Text S1), Encoding methods (Text S2), Details of data sources and methods (Text S3), Model reliability verification and CPWs setups (Text S4), The overview of the data set (Figure S1), The scenario of antibiotic emissions reduction (Figure S2), Predictive performance of RF, KNN-Boost, SVR, and KNN (Figure S3), Spatial distribution of wetland density, antibiotic removal, antibiotic surplus, and risk quotient in 2010 across China (the municipal level) (Figure S4), The distribution of field-scale CPWs across China in 2010 (Figure S5), The feature permutation importance by Eli5 (Figure S6), Comparison of ARE in various structural types of CPWs (Figure S7), Comparison of HRA in IM-WR3–60 scenarios with unenhanced and enhanced constructed wetlands (excluding reservoirs) by 2035 (Figure S8), Predictive performance of XGBoost-one-hot (Figure S9), Setups of CPWs (Figure S10), List of abbreviations defined by authors (Table S1), Empirical categories and input features in the wetland-antibiotics data set (Table S2), and The tuned hyperparameters of XGBoost, RF, KNN-Boost, SVR, and KNN models for the prediction of ARE in wetlands (Table S3) (PDF)
Wetland-antibiotics data set-Excel 1 (XLSX)
Constructed purification wetland list across China until 2020-Excel 2 (XLSX)
