# Supplementary material for: Unveiling the Role of Wetland Strategies in Antibiotic Risk Reduction across China by Machine Learning
Source: Environ Sci Technol. 2025 Jul 23;59(30):15865–76. doi: 10.1021/acs.est.5c02866 (PMC12329719; doi:10.1021/acs.est.5c02866)
Supplement: Supplementary file 1 [file es5c02866_si_001.pdf]

Supporting Information

Unveiling the Role of Wetland Strategies in Antibiotic Risk Reduction across China by Machine Learning

Lei Chen [a, b], *Junxiang Shi* [a], *Danni Wu* [a], *Ying Zhu* [c], *Jonathan M. Adams* [e], *Jichun Wu* [d], *Xiaohui Chen* [b*], *and Hongyan Guo* [a, f*]

[a] State Key Laboratory of Pollution Control and Resource Reuse, School of the Environment, Nanjing University, Nanjing 210023, China.

[b] Geomodelling and AI Centre, School of Civil Engineering, University of Leeds, Leeds LS2 9JT, UK.

[c] State Environmental Protection Key Laboratory of Environmental Health Impact Assessment of Emerging Contaminants, School of Environmental Science and Engineering, Shanghai Jiao Tong University, Shanghai 200240, China.

[d] Key Laboratory of Surficial Geochemistry of Ministry of Education, School of Earth Sciences and Engineering, Nanjing University, Nanjing 210023, China.

[e] School of Geography and Ocean Science, Nanjing University, Nanjing 210023, China.

[f] Quanzhou Institute for Environmental Protection Industry, Nanjing University, Quanzhou 362046, China.

*Corresponding author:

E-mail address: hyguo@nju.edu.cn (H. G.)

E-mail address: X.Chen@leeds.ac.uk (X. C.)

Content: 4 Texts, 10 Figures, 3 Tables, 17 Pages

Text S1. Data imputation method – missForest.

Text S2. Encoding methods.

Text S3. Details of data sources and methods.

Text S4. Model reliability verification and CPWs setups.

Figure S1. The overview of the dataset.

Figure S2. The scenario of antibiotics emissions reduction.

Figure S3. Predictive performance of (a) RF, (b) KNN-Boost, (c) SVR, and (d) KNN.

Figure S4. Spatial distribution of (a) wetland density, (b) antibiotics removal, (c) antibiotics surplus, and (d) risk quotient in 2010 across China (the municipal level).

Figure S5. The distribution of field-scale CPWs across China in 2010.

Figure S6. The feature permutation importance by Eli5.

Figure S7. Comparison of ARE in various structural types of CPWs.

Figure S8. Comparison of HRA in IM-WR3-60 scenarios with unenhanced and enhanced constructed wetlands (excluding reservoirs) by 2035.

Figure S9. Predictive performance of XGBoost-one-hot.

Figure S10. Setups of CPWs.

Table S1. List of abbreviations defined by authors.

Table S2. Empirical categories and input features in the wetland-antibiotics dataset.

Table S3. The tuned hyper-parameters of XGBoost, RF, KNN-Boost, SVR, and KNN model for the prediction of ARE in wetlands.

Supplementary Text

Text S1. Data imputation method – missForest

The missForest as an iterative imputation method was proposed by Stekhoven et al.,[1] which is based on random forests (RF). In each iteration, to impute missing values of a variable X_K^M, missForest first fits a RF with $X_K^M \sim X_{-K}^M$ using rows that do not contain missing X_K^M values, then it uses the trained RF to predict missing values in X_K^M. This process is done for all $k \in \{1, \cdots, d\}$, in the order of the number missing values in X_K^M from the fewest to the most. For the first iteration, missForest makes an initial guess for the missing values in X^M using mean imputation or another simple imputation method. The whole procedure is repeated until a stopping criterion is met.[2] By averaging many unpruned classification or regression trees, RF intrinsically constitutes a multiple imputation scheme. Using the built-in out-of-bag error estimates of random forest, one can estimate the imputation error without the need for a test set. It performs especially well in data settings where complex interactions and non-linear relations are suspected.

Text S2. Encoding methods

To ensure that these features can be recognized correctly by ML, one-hot encoding was applied to convert modified substrate and aeration into binary vectors. In addition, the digitization process of wetland structural types was further compared by ordinal encoding and one-hot encoding due to its complex four structural types.[3] Ordinal encoding for structural types is sorted according to the mean ARE of each structural type (Figure S7). After careful consideration, we selected ordinal encoding as the digitization method for wetland structural types because it favors ML to show better predictive performance in this study.

As shown in Figure S9, the train and test R^2 of model prediction by one-hot encoding structural types were 0.94 and 0.83, respectively. Although the train R^2 was 0.2 higher than it by the ordinal encoding method (Figure 1a), the test R^2 decreased by 0.1. This is due to the fact that the dimension of input parameters increases when the one-hot encoding process is used for the digitization process of structural types, thus increasing the complexity of the model. For small datasets, too high model complexity will increase the risk of overfitting, resulting in a larger gap between train R^2 and test R^2, which is not conducive to model prediction. To sum up,

based on the results of gap between train R^2 and test R^2, we choose ordinal encoding, which has less influence on model prediction, as a digitization method for structural types.

Text S3. Details of data sources and methods

We used the publicly available, two national land resource survey datasets (https://gtdc.mnr.gov.cn/) conducted in China in 2010 and 2020 to create the wetland distribution map. According to the wetland classification national standard (GB/T 24708-2009), we classified the types of wetlands into natural wetlands (including riverine wetlands, lacustrine wetlands, marshy wetlands and coastal wetlands) and constructed wetlands (including reservoirs, channels and ponds).

In this study, we use a recently established veterinary antibiotic emissions dataset in a best-guess emission scenario, calculated at the county level in China, to assess spatially varying antibiotics inputs to wetlands, assuming no process loss.[4] The veterinary antibiotic emissions were selected as a representation for antibiotic inputs on account of two reasons: (i) veterinary antimicrobials account for approximately 73% of global antimicrobials consumption;[5] (ii) The discharge percentage of human antibiotics is less than 8% in the water environment due to the limitation of consumption.[6] It should be emphasized that the calculation of antibiotics input in a best-guess scenario has incorporated the influence of pretreatment processes (e.g. manure treatment processes) and use policy. For additional details, see Li et al.[4]

The annual average air temperature variable at the county level was obtained from the China Surface Climate Dataset V3.0 (http://data.cma.cn/). The water quality property values (including pH, COD, TP, TN and NH_4^+-N) were obtained using data from the China National Environmental Monitoring Centre (https://www.cnemc.cn/). The average annual monitoring value of the water quality section at the municipal level is used as the input value of the model water quality characteristics of the region. Refer to the average recommended planting density in various wetland types from the Technical specification for wetland ecological restoration of China (LY/T 3353-2023 and DB 43/T 2942-2024), the input values (plants/m^2) of planting density were 6.86, 7.87, 10.33, 15.85 and 7.56 for riverine wetland, lacustrine wetland, marshy wetland, coastal wetland and constructed wetlands, individually. The input value of the wetland depth was selected as 2 m based on the Classification of Wetlands and Deepwater Habitats of the United States.[7] We regard the wetland in a single county area as a hybrid wetland whole

based on the wetland connectivity.[8] County-scale antibiotic inputs were fully and equally allocated to wetlands within the region. Considering the regional variation of HRT, they were set between 2-10 d. Since the RQ value calculation is concentration dependent and further HRT dependent, we fixed the HRT at 5 days in risk assessment. The antibiotics concentration c (in mg/L) in a single type of wetland at the county scale was estimated using eq. S1.

$$c_i = (AI_i * HRT_i)/(Operation\ time * Depth * Aera_i) \quad \text{(S1)}$$

The proportion of different antibiotic types was obtained from the antibiotics emission dataset.[4] The other input parameters like operation time, modified substrate and aeration were fixed as 365 days, unmodified substrate and non-aeration, respectively.

Text S4. Model reliability verification and CPWs setups

A small external validation dataset (a total of 7 data points was independent of the initial wetland-antibiotics dataset) was established using as same as collection method described in the wetland-antibiotics dataset from June 2023 to October 2023 (Supplementary Data—Excel 1). The XGBoost model was used to predict ARE in the external validation dataset.

Meanwhile, we tested CPWs with quartz sand substrate as experiment validation for exploring trained model reliability. The structural type of CPWs were set as the vertical subsurface flow type.[9] Our CPW experiment was in duplicate. CPWs were planted with *Iris* (56 plants/m^2). The cylindrical CPWs has a diameter of 0.3 m and a height of 0.5 m. The material is plexiglass and the wall thickness of the devices is 0.01 m. The water inlet on every device is located at a height of 0.05 m. After adding quartz sand into the devices, their height reached about 0.45 m. There were 180 mg/L COD ($C_6H_{12}O_6$), 30 mg/L NH_4^+-N (NH_4Cl), 7.3 mg/L $CaCl_2 \cdot 2H_2O$, 4.5 mg/L $MgSO_4 \cdot 7H_2O$, 0.05 mg/L $FeCl_3$, 17.5 mg/L KH_2PO_4, 0.2 mg/L sulfamethazine (SMZ) in feed water. The HRT of CPWs was for 2 days in the formal operation. The inlet and outlet at the lower and upper parts of the CPW setup were connected to the peristaltic pump, respectively. Each CPWs device was wrapped with a shading cloth (Figure S10). The laboratory environment temperature was controlled at 25 ℃ to the greatest extent. The pH of inflow was adjusted around 6.

All chemicals and reagents used as received without further purification in this research, and were in analytical grade or higher. Sulfamethazine (SMZ, 99.5%) was obtained from Shanghai Titan Scientific Co., Ltd. (Shanghai, China). The SMZ measuring mobile phase

consisted of acetonitrile/water (35:65, v/v) wavelength a flow rate of 1.0 mL·min^{-1} and 266 nm wavelength by high performance liquid chromatography (Agilent 1260, USA).[9]

Supplementary Figures

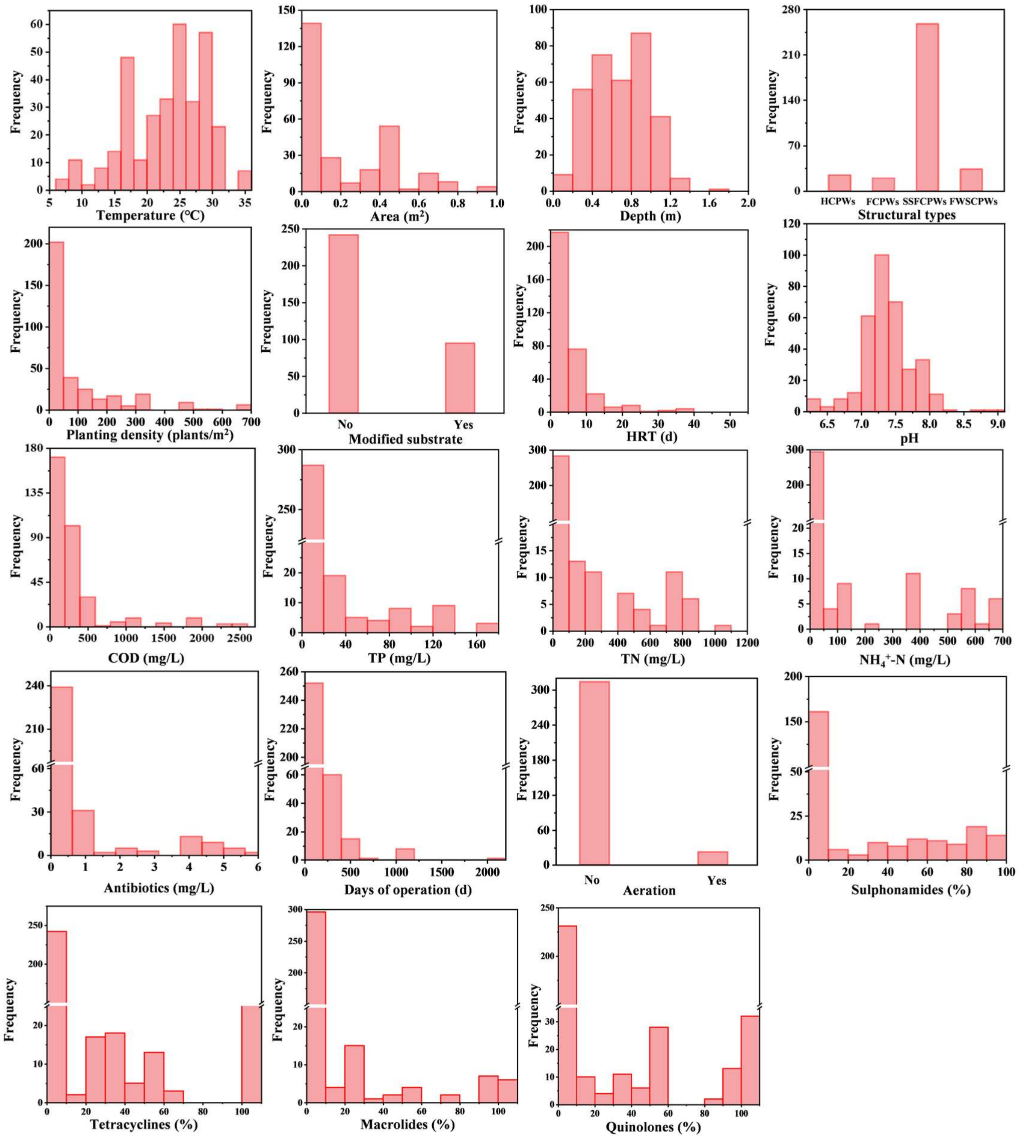

Figure S1. The overview of the dataset, containing 337 instances, used for ML. Due to the data distribution range being too large, only 85.2% of Area data and 91.7% of antibiotics data are shown in Figure S1.

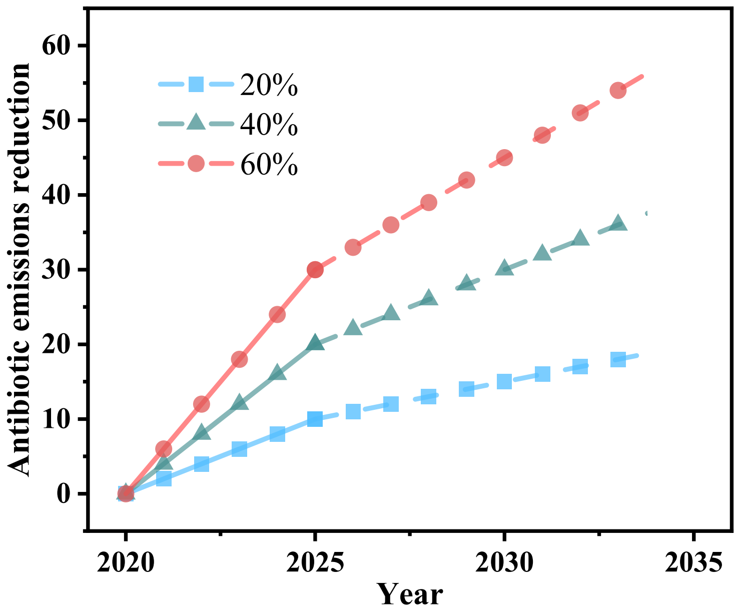

Figure S2. The scenario of antibiotics emissions reduction.

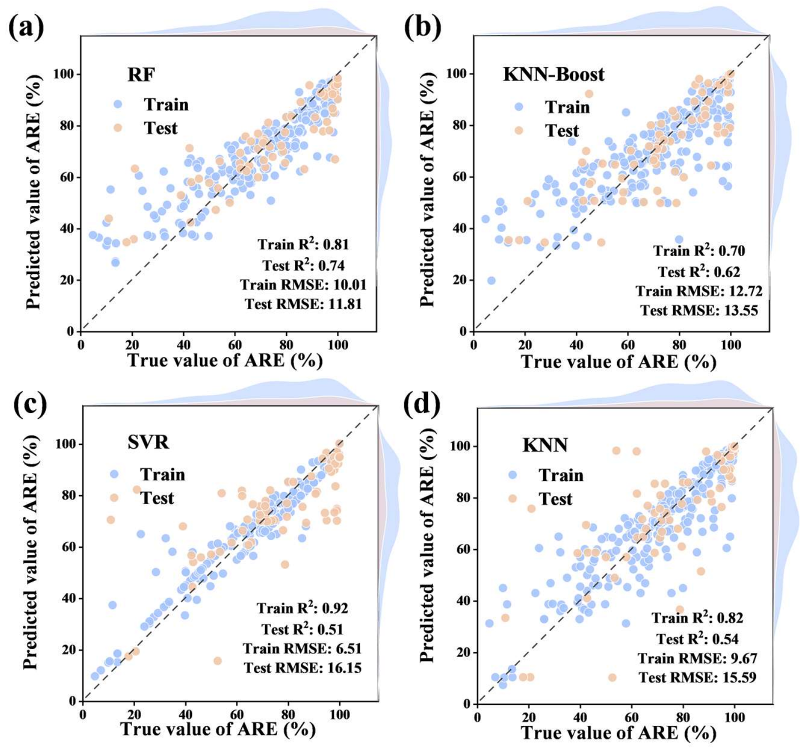

Figure S3. Prediction performance of (a) RF, (b) KNN-Boost, (c) SVR, and (d) KNN. The gray dashed lines indicate the line of equality (predicted value = true value).

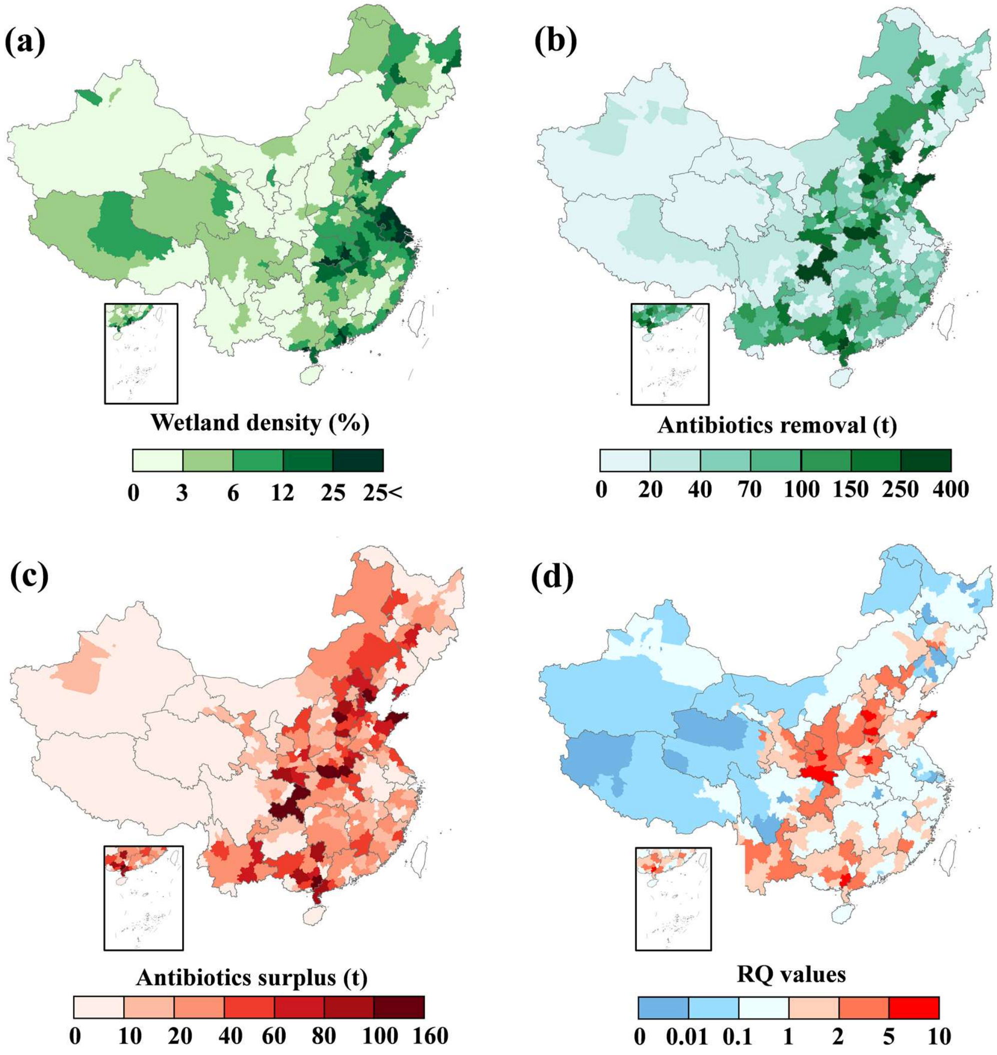

Figure S4. Spatial distribution of (a) wetland density, (b) antibiotics removal, (c) antibiotics surplus, and (d) risk quotient in 2010 across China (the municipal level).

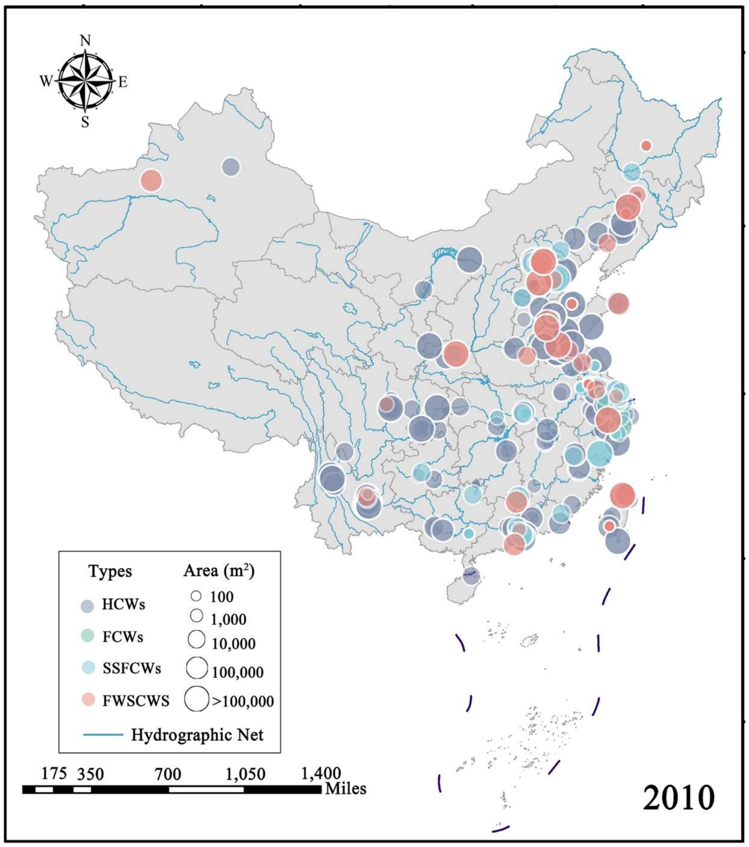

Figure S5. The distribution of field-scale CPWs across China in 2010.

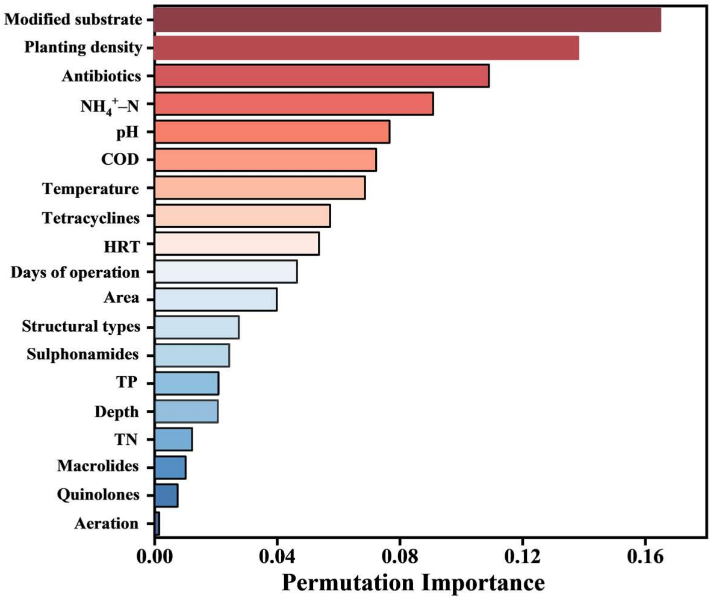

Figure S6. The feature permutation importance by Eli5.

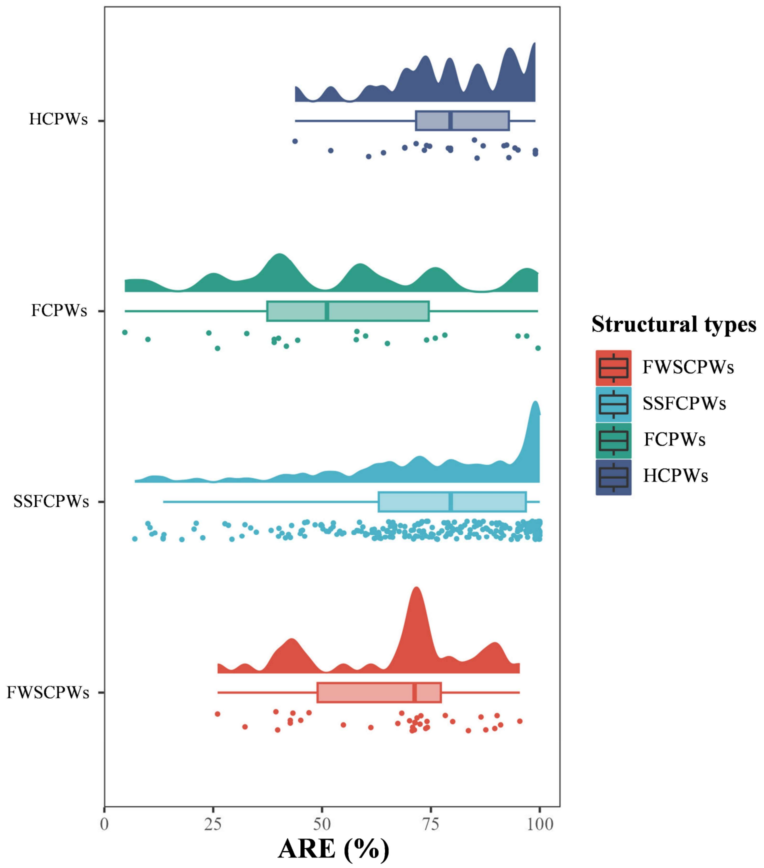

Figure S7. Comparison of ARE in various structural types of CPWs. The hybrid CPWs (HCPWs) present the best mean ARE (80.4%), and it in the subsurface flow CPWs (SSFCPWs) (75.4%) was better than in the free water surface flow CPWs (FWSCPWs) (66.4%) and floating CPWs (FCPWs) (53.1%).

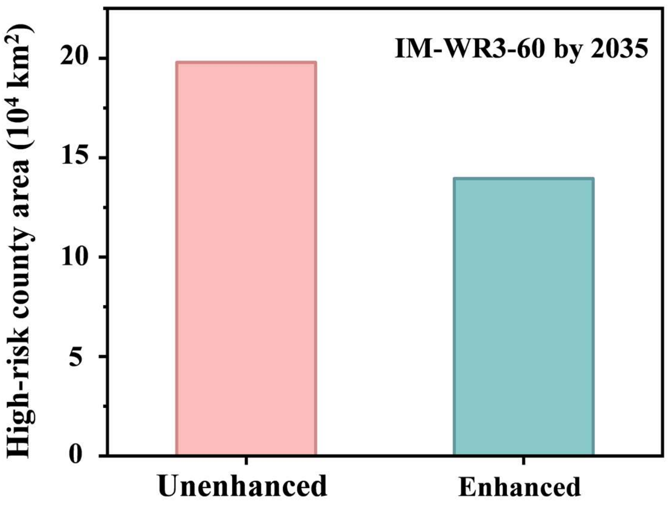

Figure S8. Comparison of HRA in IM-WR3-60 scenarios with unenhanced and enhanced

constructed wetlands (excluding reservoirs) by 2035.

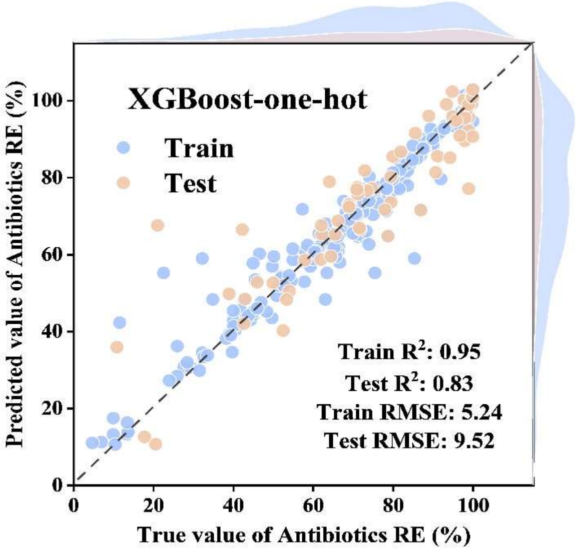

Figure S9. Predictive performance of XGBoost-one-hot. The gray dashed lines indicate the line of equality (predicted value = true value).

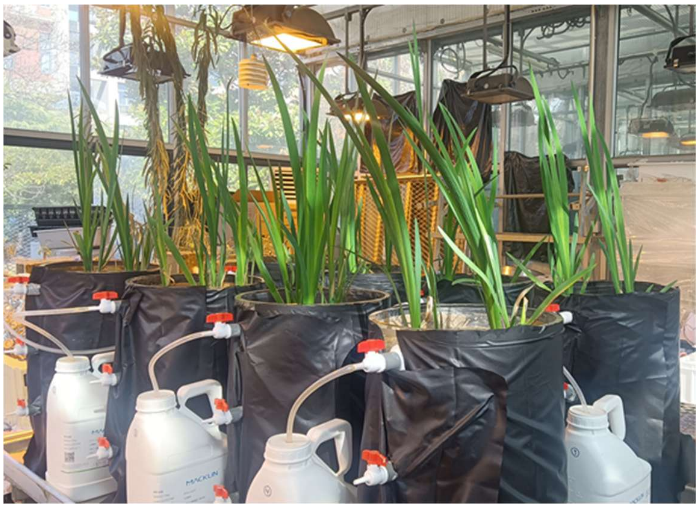

Figure S10. Setups of CPWs.

Supplementary Tables

Table S1. List of abbreviations defined by authors

Abbreviations	Meaning
CPWs	Constructed purification wetlands
ARE	Antibiotics removal efficiency
ML	Machine learning
SHAP	Shapley Additive exPlanations
HRT	Hydraulic retention time
XGBoost	Extreme gradient boosting
KNN	K-nearest neighbors
RF	Random forest
SVR	Support vector regression
DT	Decision tree
R^2	Coefficient of determination
RMSE	Root mean square error
FCPWs	Floating CPWs
FWSCPWs	Free water surface flow CPWs
SSFCPWs	Subsurface flow CPWs
HCPWs	Hybrid CPWs
TP	Total of phosphorus
TN	Total of nitrogen
COD	Chemical oxygen demand
RQ	Risk quotient
HRA	High-risk county area
IM	Improved management
WR	Wetland restoration
AR	Antibiotics reduction

Table S2. Empirical categories and input features in the wetland-antibiotics dataset

Empirical categories	Input Features	Description	Unit	Data range
Wetland construction characteristics	Area	Wetland area	m^2	0.003-90000
	Depth	Wetland depth	m	0.1-1.7
	Structural types	FCPWs (1); FWSCPWs (2); SSFCPWs(3); HCPWs (4)	/	1/2/3/4
Hydraulic conditions and temperature	HRT	Hydraulic retention time	d	0.15-50
	Days of operation	Wetland operation time	d	5-2160
	Temperature	Environmental temperature	℃	6-35
Water quality properties	pH	The pH value of influent	/	6.35-9.07
	COD	The chemical oxygen demand of influent	mg/L	0-2425
	TP	The total phosphorus concentration of influent	mg/L	0-161
	TN	The total nitrogen concentration of influent	mg/L	0-1086
	NH_3–N	The ammonia nitrogen concentration of influent	mg/L	0-679
	Antibiotics	The antibiotics concentration of influent	mg/L	0.00000096-100
	Sulphonamides	The sulphonamides proportion of antibiotics	%	0-100
	Tetracyclines	The tetracyclines proportion of antibiotics	%	0-100
	Macrolides	The macrolides proportion of antibiotics	%	0-100
	Quinolones	The quinolones proportion of antibiotics	%	0-100
Artificial configuration parameters	Planting density	Planting density of wetland	plants/m^2	0-667
	Modified substrate	Adopting modified substrate or not	Yes/No	1/0
	Aeration	Adopting aeration or not	Yes/No	1/0

Note: In this study, the modified substrate was defined as a category of substrates that are modified through physical, chemical, or biological methods to improve pollutants removal performance in wetlands. For example, biochar, modified ore, microbial fuel cell, active microorganisms modification and so on.

Table S3. The tuned hyper-parameters of XGBoost, RF, KNN-Boost, SVR, and KNN model for the prediction of ARE in wetlands

Prediction target	ML models	Hyper-parameters (three vital parameters)		
Antibiotics removal efficiency	XGBoost	n_estimators	max_depth	colsample_bytree
		215	16	0.7
	RF	n_estimators	max_depth	min_samples_split
		172	33	1
	KNN-Boost	n_estimators	n_neighbors	learning_rate
		22	3	0.044
	SVR	C	epsilon	coef0
		1980	5	12
	KNN	n_neighbors	leaf_size	p
		2	74	1

References

1. Stekhoven, D. J.; Buhlmann, P., MissForest--non-parametric missing value imputation for mixed-type data. Bioinformatics **2012**, 28, (1), 112-8.

2. Sun, Y.; Li, J.; Xu, Y.; Zhang, T.; Wang, X., Deep learning versus conventional methods for missing data imputation: A review and comparative study. Expert Syst. Appl. **2023**, 227, 120201.

3. Xu, P.; Li, G.; Zheng, Y.; Fung, J. C. H.; Chen, A.; Zeng, Z.; Shen, H.; Hu, M.; Mao, J.; Zheng, Y.; Cui, X.; Guo, Z.; Chen, Y.; Feng, L.; He, S.; Zhang, X.; Lau, A. K. H.; Tao, S.; Houlton, B. Z., Fertilizer management for global ammonia emission reduction. Nature **2024**, 626, (8000), 792-798.

4. Li, S.; Zhu, Y.; Zhong, G.; Huang, Y.; Jones, K. C., Comprehensive Assessment of Environmental Emissions, Fate, and Risks of Veterinary Antibiotics in China: An Environmental Fate Modeling Approach. Environ. Sci. Technol. 2024, 58, (12), 5534-5547.

5. Van Boeckel, T. P.; Pires, J.; Silvester, R.; Zhao, C.; Song, J.; Criscuolo, N. G.; Gilbert, M.; Bonhoeffer, S.; Laxminarayan, R., Global trends in antimicrobial resistance in animals in low- and middle-income countries. Science **2019**, 365, eaaw1944.

6. Chen, Y.-R.; Duan, Y.-P.; Zhang, Z.-B.; Gao, Y.-F.; Dai, C.-M.; Tu, Y.-J.; Gao, J., Comprehensive evaluation of antibiotics pollution the Yangtze River basin, China: Emission, multimedia fate and risk assessment. J. Hazard. Mater. **2024**, 465, 133247.

7. Cowardin, L. M.; Golet, F. C., US-FISH-AND-WILDLIFE-SERVICE 1979 WETLAND CLASSIFICATION - A REVIEW. Vegetatio 1995, 118, (1-2), 139-152.

8. Leibowitz, S. G.; Hill, R. A.; Creed, I. F.; Compton, J. E.; Golden, H. E.; Weber, M. H.; Rains, M. C.; Jones, C. E., Jr.; Lee, E. H.; Christensen, J. R.; Bellmore, R. A.; Lane, C. R., National hydrologic connectivity classification links wetlands with stream water quality. Nat. Water 2023, 1, 370-380.

9. Chen, L.; Wu, D.; Jiang, T.; Yin, Y.; Du, W.; Chen, X.; Sun, Y.; Wu, J.; Guo, H., A novel heterogeneous catalytic system (AC/ZVI/CaO_2) promotes simultaneous removal of phosphate and sulfamethazine: Performance, mechanism and application feasibility verification. Water Res. 2023, 237, 119977.

Data S1. (separate file)

Excel 1-Wetland-antibiotics dataset

Data S2. (separate file)

Excel 2-Constructed purification wetland list across China until 2020
